# Nucleic acid joining enzymes: biological functions and synthetic applications beyond DNA

**DOI:** 10.1042/BCJ20240136

**Published:** 2025-01-22

**Authors:** Chelsea Blackstock, Caitlin Walters-Freke, Nigel Richards, Adele Williamson

**Affiliations:** 1 School of Science, University of Waikato, Hamilton, Waikato, 3216, New Zealand; 2 Foundation for Applied Molecular Evolution, Alachua, FL, 32615, U.S.A; 3 School of Chemistry, Cardiff University, Cardiff, CF10 3AT, U.K

**Keywords:** artificially expanded genetic information systems, DNA ligase, DNA polymerase, nucleic acid synthesis, xenobiotic nucleic acid

## Abstract

DNA-joining by ligase and polymerase enzymes has provided the foundational tools for generating recombinant DNA and enabled the assembly of gene and genome-sized synthetic products. Xenobiotic nucleic acid (XNA) analogues of DNA and RNA with alternatives to the canonical bases, so-called ‘unnatural’ nucleobase pairs (UBP-XNAs), represent the next frontier of nucleic acid technologies, with applications as novel therapeutics and in engineering semi-synthetic biological organisms. To realise the full potential of UBP-XNAs, researchers require a suite of compatible enzymes for processing nucleic acids on a par with those already available for manipulating canonical DNA. In particular, enzymes able to join UBP-XNA will be essential for generating large assemblies and also hold promise in the synthesis of single-stranded oligonucleotides. Here, we review recent and emerging advances in the DNA-joining enzymes, DNA polymerases and DNA ligases, and describe their applications to UBP-XNA manipulation. We also discuss the future directions of this field which we consider will involve two-pronged approaches of enzyme biodiscovery for natural UBP-XNA compatible enzymes, coupled with improvement by structure-guided engineering.

## Introduction

Enzyme-mediated cutting, joining and amplification of canonical DNA using restriction endonucleases, DNA ligases and polymerases to generate recombinant DNA are the foundational tools of modern molecular biology. Innovations in both the synthesis and assembly steps have led to the synthetic biology revolution, paving the way for semi-synthetic or fully synthetic organisms and other bio-inspired technologies of the future [1,2]. The development of highly efficient synthesis of user-defined polynucleotide sequences has applications ranging from commercial provision of primers and probes bearing fluorescent labels and other functional group modifications to construction of bespoke gene sequences incorporating specific mutations, reading-frame fusions and optimised codons for improved heterologous protein expression [3].

**Figure 1:**
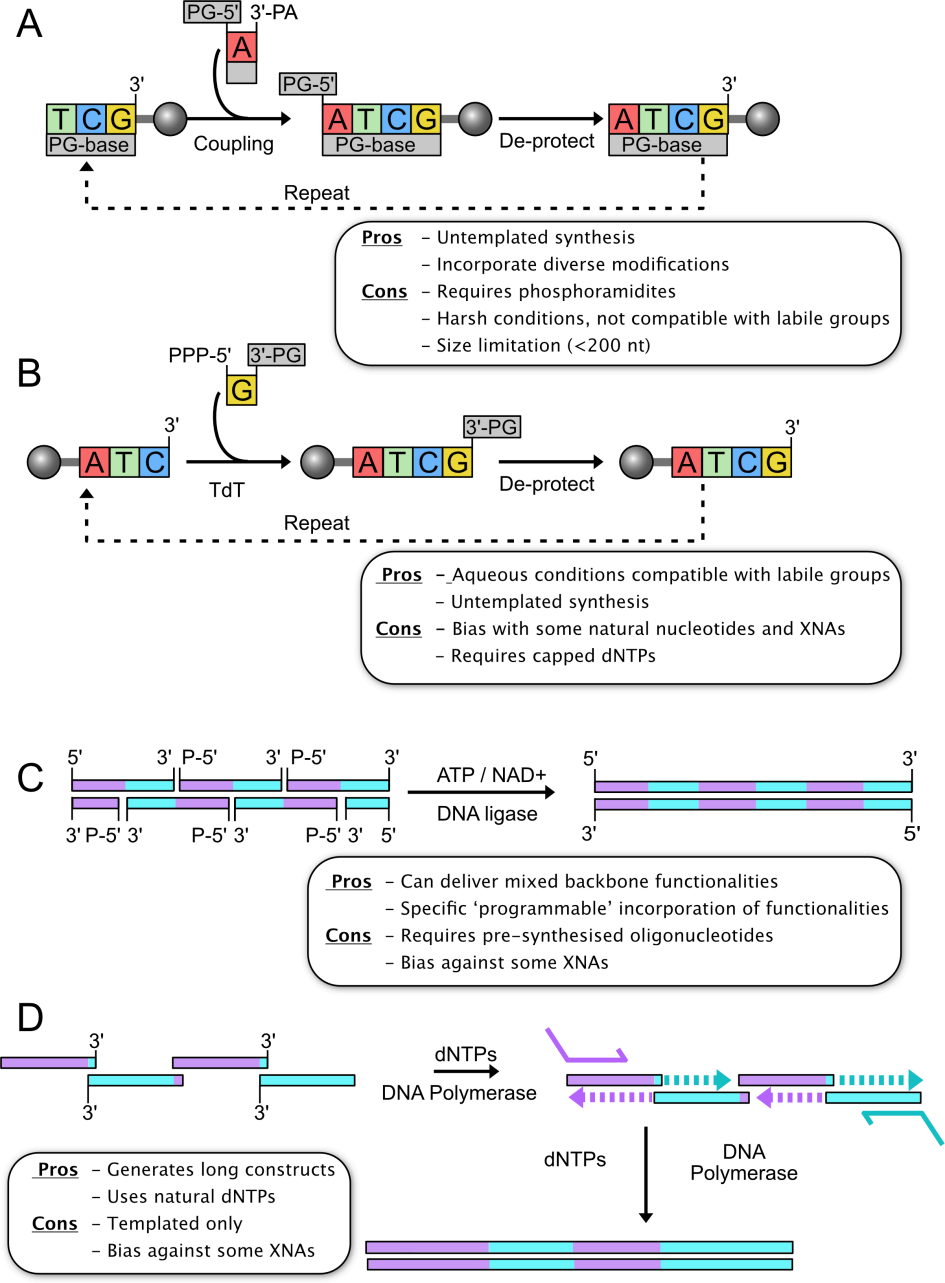
Schematic of different DNA synthesis and assembly methods. (**A**) The phosphoramidite synthesis method generates single-stranded nucleic acid by sequentially adding 2′ deoxy nucleoside phosphoramidites via the reactive 3′ functionality (3′ PA) to the 5′ OH terminus of the chain, which is immobilised on a bead. Protecting groups prevent unwanted side -reactions on the 5′ OH terminus of the incoming nucleoside phosphoramidite (PG-5′) and on OH and NH_2_ groups of the bases (PG-Base). The deprotection step subsequent to nucleoside phosphoramidite addition generates a free 5′ OH terminus for the next extension step. (**B**) Terminal deoxy nucleotidyl transferase (TdT) synthesis appends single 2′ deoxy ribonucleotide triphosphates (dNTPs) to the 3′ terminus of the immobilised oligonucleotide chain in an enzyme-catalysed step. To ensure only one dNTP is added per extension cycle, the 3′ OH of the incoming dNTP is protected (3′’PG) and this cap is removed in a deprotection step prior to the next addition to generate a free 3′ terminus. (**C**) DNA-ligase- mediated assembly uses short (<20 nt) pre-synthesised oligonucleotides with complementary overhangs that generate a contiguous nicked duplex with no gaps. All oligonucleotides at internal positions are 5′ phosphorylated, and the DNA ligase joins these directly to the adjacent 3′ OH terminus to generate the double-stranded product. (**D**) Polymerase-based synthesis/ assembly begins with a series of oligonucleotides with short cohesive overlaps that anneal to give a non-contiguous duplex with long stretches of single-stranded nucleic acid. In the first step, the polymerase extends from the 3′ OH of the acceptor strand by the addition of dNTPs, using the complementary strand as a template. Successive cycles of melting, annealing and extension create a complete duplex which spans the entire range and is amplified with external primers in a final step.

Currently, the most common method for *de novo* polynucleotide synthesis at commercial scale uses nucleoside phosphoramidite chemistry, which has a long and well-documented history and has been the subject of extensive reviews [4–6]. The typical solid-phase approach involves the addition of one 5′-protected dimethoxytrityl (DMT) nucleotide phosphoramidite at a time to a growing strand, that is attached to a solid support (Figure 1A). Use of microarrays increases throughput by enabling the simultaneous synthesis of numerous diverse oligonucleotide strands. However, solid-phase synthesis is limited to products less than 200 nucleotides (nt) in length, because side reactions occurring in each addition cycle negatively impact yields [7]. On the other hand, as reviewed recently [8], enzymatic approaches for the production of longer oligos have proved to be an effective solution to this problem. Template-independent enzymatic oligonucleotide synthesis (TiEOS) methods generate oligonucleotides using the terminal deoxynucleotidyl transferase (TdT) polymerase which incorporates 3′-protected nucleoside 5′ triphosphates (NTPs) onto the 3′-terminus of DNA possessing a 3′-overhang (Figure 1B). The use of 3′-capped dNTPs enables the controlled introduction of nt to build sequence-specific oligomers [9]. Additional strategies to generate extended duplexes may be ligation based, where shorter oligonucleotides are synthesised with complementary overhangs and then joined together using a ligase enzyme such as T4 DNA ligase. Alternatively, polymerase cycling assembly, in which overlapping synthetic oligonucleotides are amplified using a DNA polymerase, can produce long pieces of double-stranded DNA (Figure 1C and D) [6]. Both these latter methods, however, rely on base pairing and provision of pre-synthesised oligonucleotides and so do not constitute entirely *de novo* nucleic acid synthesis; however, they can be used to build or amplify larger constructs using oligonucleotides generated from the first two methods.

**Figure 2:**
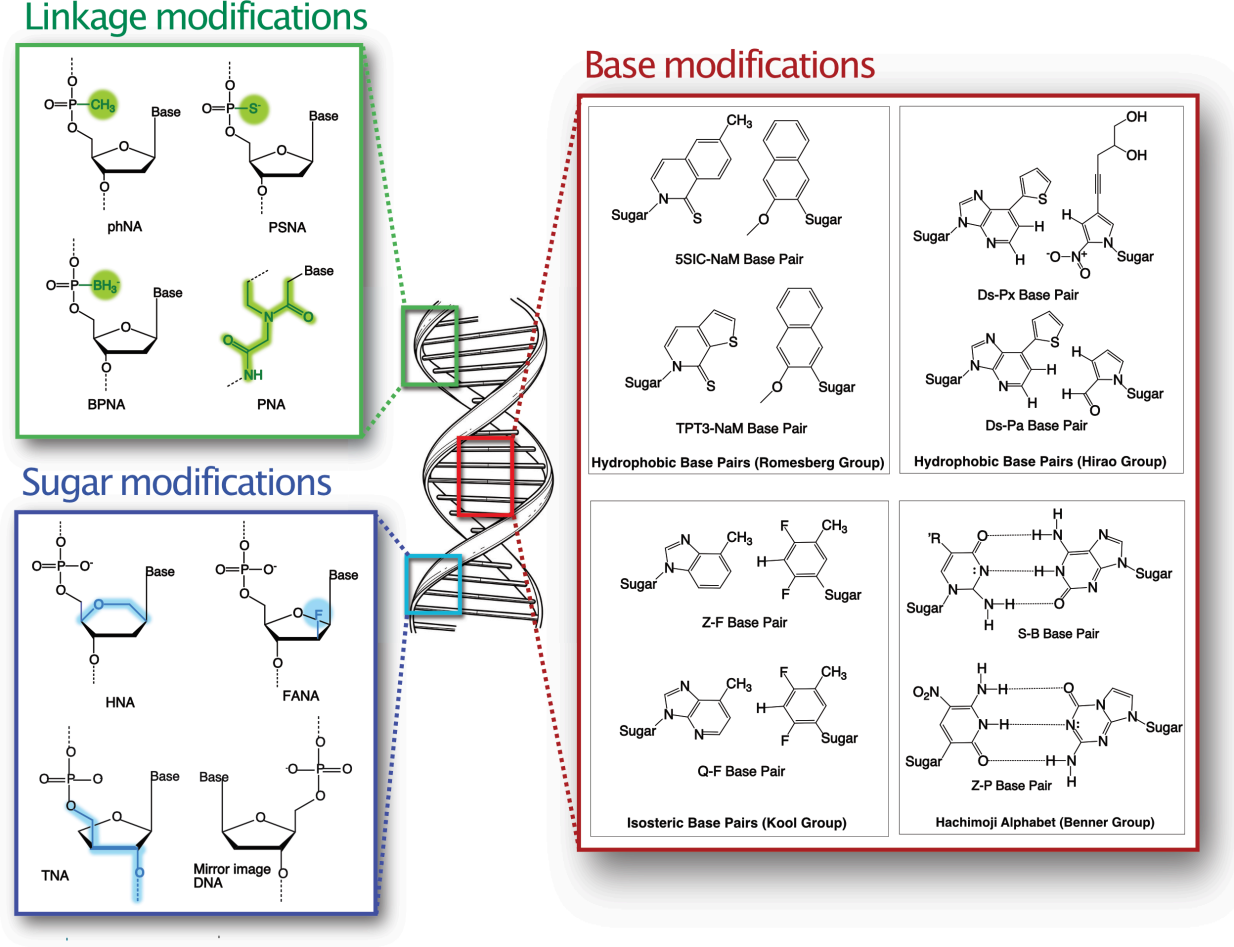
Representative examples of different XNAs. Linkage modifications substitute of one of the non-bridging oxygens in the phosphodiester backbone, or replace this backbone altogether. In alkyl phosphonate nucleic acid (phNA) this substitution is a methyl group, in phosphorothioate nucleic acid (PS) it is a sulphur and in boranophosphate (BPNA) it is a borano group. The peptide nucleic acid (PNA) has a peptide linkage in place of the phosphodiester bond. Sugar modifications include replacement of the 2′ oxygen with fluorine in 2′′-fluoro arabinonucleic acid (FANA), or substitution with an alternate sugar as in hexitol nucleic acid (HNA) where 1,5-anhydrohexitol replaces ribose, or threose nucleic acid (TNA) where threose is substituted. In mirror -image DNA, the d-form of the ribose is replaced with the l-ribose enantiomer. Base modifications include substitution of the hydrogen-bonded nitrogenous bases with moieties that pair through hydrophobic interactions, or with isosteres of the natural nitrogenous bases that lack the hydrogen bonds but interact through shape complementarity. The Hachimoji pairs include pyrimidine/purine equivalents of the natural bases, but with rearranged hydrogen bonding patterns.

An emerging area of nucleic acid research is the use of xenobiotic nucleic acids (XNAs) in synthetic biology, therapeutic and biosensing applications [10]. XNAs are nucleic acid analogues of DNA and RNA which have non-natural substitutions in their sugar moiety, phosphodiester linkages or base pairs (Figure 2) [11]. The exact definition of the abbreviation ‘XNA’ as a general umbrella term for non-biological alternatives to canonical DNA and RNA is currently a matter of discussion. Debate centres on the use of more specific identifiers to delineate the location of the non-canonical moiety (e.g. sugar vs. base) and whether chemically modified variations of natural nucleic acids (e.g. 2′OMe-RNA) should be included. For the purposes of this review article, we use XNA to include all nucleic acids with non-natural substitutions. For a more nuanced account of the proposed redefinitions, the reader is directed to Chaput and Herdewijn [12].

Many XNA applications will eventually require enzymes to produce extended polynucleotide assemblies at the gene, plasmid or even genomic scales. Modern molecular biologists are spoilt by the range of enzymes available for amplification and assembly of natural DNA and RNA nucleic acids. These enzymes have been sourced from the replication, recombination and repair machinery of cellular organisms and viruses, and in many cases, their *in vitro* activity has been improved either by optimising reaction conditions, or by enzyme engineering [13,14]. Currently, the majority of XNA-containing oligonucleotides are synthesised using phosphoramidite methods. In addition to the usual intrinsic limitations on length, this strategy is complicated by the chemical instability of some functionalities and the lack of available phosphoramidite precursors with appropriate protecting groups. Enzymatic synthesis of XNA polynucleotides presents a possible solution to this issue because the mild reaction conditions, typically aqueous and neutral pH, are compatible with labile functional groups. Unfortunately, many of the widely available enzymes used for natural DNA amplification and assembly are only partially functional on XNA substrates and there is typically a compromise in activity, fidelity or a bias against the non-canonical substrate [15–19].

This review summarises the use of different DNA polymerases and DNA ligases in nucleic acid synthesis and their potential for the generation and assembly of XNA-containing oligonucleotides. Ligases and polymerases are both found in structurally and functionally diverse classes, and we begin with a brief account of the biological role of each enzyme class followed by their utility in biotechnology. We then describe recent research into the compatibility of each enzyme type with technologically relevant categories of XNAs that contain non-natural base pairs which are orthogonal to those in canonical DNA. Finally, we discuss future directions in enzyme-mediated XNA synthesis and the role that novel and/or engineered proteins may play in this.

## Unnatural base pair xenobiotic nucleic acids: beyond canonical bases and genetic alphabets

XNAs are analogues of DNA or RNA which have alternative chemical structures in either the sugar-phosphate backbone, or the nucleobases. Non-canonical bases capable of pairing with their natural counterparts have been successfully incorporated into cellular genomes [20,21]. Others have played a pivotal role in the success of mRNA technology [22,23] and key modifications in this field are depicted in Supplementary Figure S1. Where non-canonical bases are able to form novel interactions that are mutually exclusive to natural bases, they are known as unnatural base pairs (UBPs) [24]. The desirable properties of a UBP are that the interaction is both selective and orthogonal, meaning that UBP nt partner exclusively with one another and have minimal mismatches with other canonical bases. In one of several approaches, the creation of hydrogen-bonded UBPs has allowed for the development of Artificially Expanded Genetic Information Systems (AEGIS) in which the genetic alphabet which consists of the canonical A:T/U and G:C base pairs and additional orthogonal base pairs [25,26]. At present, UBPs have been developed which can function in a similar manner to canonical base pairs during replication, transcription and translation, potentially increasing the information content which is able to be encoded in the genes of living organisms [27]. In addition to synthetic biology, other potential applications for these UBP-XNA substrates include polymer-based nanotechnology [28], incorporation into molecular beacons [29] and inclusion in single-stranded nucleic acid aptamers, which bind to targets and can be utilised in drug delivery and therapy [30–33]. Such aptamers incorporating AEGIS bases have been generated using systematic evolution of ligands by exponential enrichment (SELEX). SELEX to generate DNA aptamers involves creating a single-stranded DNA library, then selecting and enriching for particular aptamer sequences which can bind to the molecule [34]. This method has now been used to create aptamers that include non-canonical bases which have increased information density in the target binding region [35]. Such aptamers have been used to develop aptamers against targets such as anthrax protective antigen and T-cell receptor-CD3ε and have been used for prodrug delivery to cancer cells [33,36,37].

In lifeforms with artificially expanded genetic alphabets, these UBPs must be recognised by enzymatic machinery for efficient replication and transcription, while to have potential in aptamer generation, they must be able to create and maintain stable secondary structures. UBPs that fulfil these criteria fall broadly into three categories depending on how this orthogonality is achieved: rearrangement of the pattern of hydrogen bond donor–acceptor pairs in the bases, introduction of novel pairing interactions through hydrophobicity, or introduction of orthogonal interactions by non-hydrogen-bonded structural complementarity (Figure 2). Of these three broad UBP categories, novel hydrogen-bonded systems hold considerable promise as they preserve Watson-and-Crick like behaviour of natural DNA, are evolvable during replication, and the inclusion of multiple consecutive hydrogen-bonded UBPs has minimal impact on duplex properties [38,39]. Early examples of hydrogen bonding UBPs include isoG (6-amino-2-ketopurine) and isoC (2-amino-4-ketopyrimidine) which are recognised by natural enzymatic machinery in both DNA replication and transcription [40]. The isoG:isoC UBP has been successfully used to encode the unnatural amino acid 3-iodotyrosine, which was incorporated into a peptide by *in vitro* translation using a novel isoGUC anticodon system [41]. Subsequent research has resulted in improvements to the chemical stability of isoC, as well as modification to remove the minor tautomeric enol form of isoG which mispairs with thymine [42]. The synthesis of two additional hydrogen-bonding bases ‘Z’ (6-amino-3-(1′-β-d-2′-deoxyribofuranosyl)-5-nitro-1*H*-pyridin-2-one), and ‘P’ (2-amino-8-(1′-β-d-2′-deoxyribofuranosyl)-imidazo-[1,2a]-1,3,5-triazin-[8*H*]-4-one) has given rise to the 8-letter Hachimoji alphabet which includes the two non-natural S:B and P:Z UBPs in addition to the A:T and G:C pairs [17]. Duplexes of deoxyribose Hachimoji UBP-XNA exhibit similar thermostability and structural properties to natural DNA, and in an early proof-of-concept were able to be transcribed by RNA polymerase to generate a variant of the spinach fluorescent RNA aptamer which incorporated Hachimoji-base ribonucleotides [39].

Enzymatic replication and crucially biological retention have been achieved with the hydrophobic base pair dNaM-dTPT3 (2-methoxynaphthalene and thieno[2,3 *c*]pyridine-7(6*H*)-thione). This was achieved by co-expression of transporters facilitating the uptake of the non-natural dNTP into the host cells as well as CRISPR-Cas-based elimination of cells which lose the UPB-containing plasmid [43]. The dNaM-dTPT3 UBP is an analogue of the dNaM-d5SICS (6-methyl-2*H*-isoquinoline-1-thione) UBP which was modified to improve polymerase incorporation, highlighting the critical importance of compatibility between non-natural nucleobases and enzymatic replication machinery [44,45]. A second hydrophobic pair ‘DS’ (7-(2-thienyl)imidazo[4,5-*b*]pyridine) and ‘PX’ (2-nitro-4-propynylpyrrole) which bears little structural resemblance to dNaM, dTPT3 and d5SICS is also able to be replicated with high fidelity and this UBP is retained during multiple rounds of PCR which has allowed its incorporation into aptamers using SELEX [46]. As with other UBPs, the DS-PX pair represents the outcome of an iterative design process which eliminated mispairings between the novel hydrophobics and the natural bases, while retaining optimal geometry compatible with enzymatic propagation [27].

The current review focuses on development of enzymes which are compatible with the UBP-class of XNAs as this is an area where innovation in molecular tools is required, while research on enzymes which function with sugar-modified XNAs is, at present, more complete. The inherent nuclease-resistance of backbone-modified XNAs together with the enhanced duplex-forming properties of many of these variants make them extremely attractive for biotechnology; especially as bio-stable therapeutics [10,11]. As sugar-modified XNAs and their manipulation have been the subject of several excellent reviews in recent years [47–50], they will not be discussed in detail here; however, we point the reader to several key examples where compatibility of polymerase and ligase with backbone-modified XNA has been established [51].

## Nucleic acid polymerases

### Classes of DNA polymerase and their biological functions

DNA polymerases catalyse nucleic acid polymer synthesis by the addition of deoxyribonucleotide triphosphates (dNTPs) to the 3′ terminus of a DNA or RNA primer [52]. In almost all cases, synthesis of the new strand is directed by the presence of a complementary antiparallel strand (template) which determines the insertion of new nt based on Watson–Crick base-pairing rules. However, as discussed below, some polymerases are capable of non-templated addition of nt to the 3′ end of the primer.

**Figure 3:**
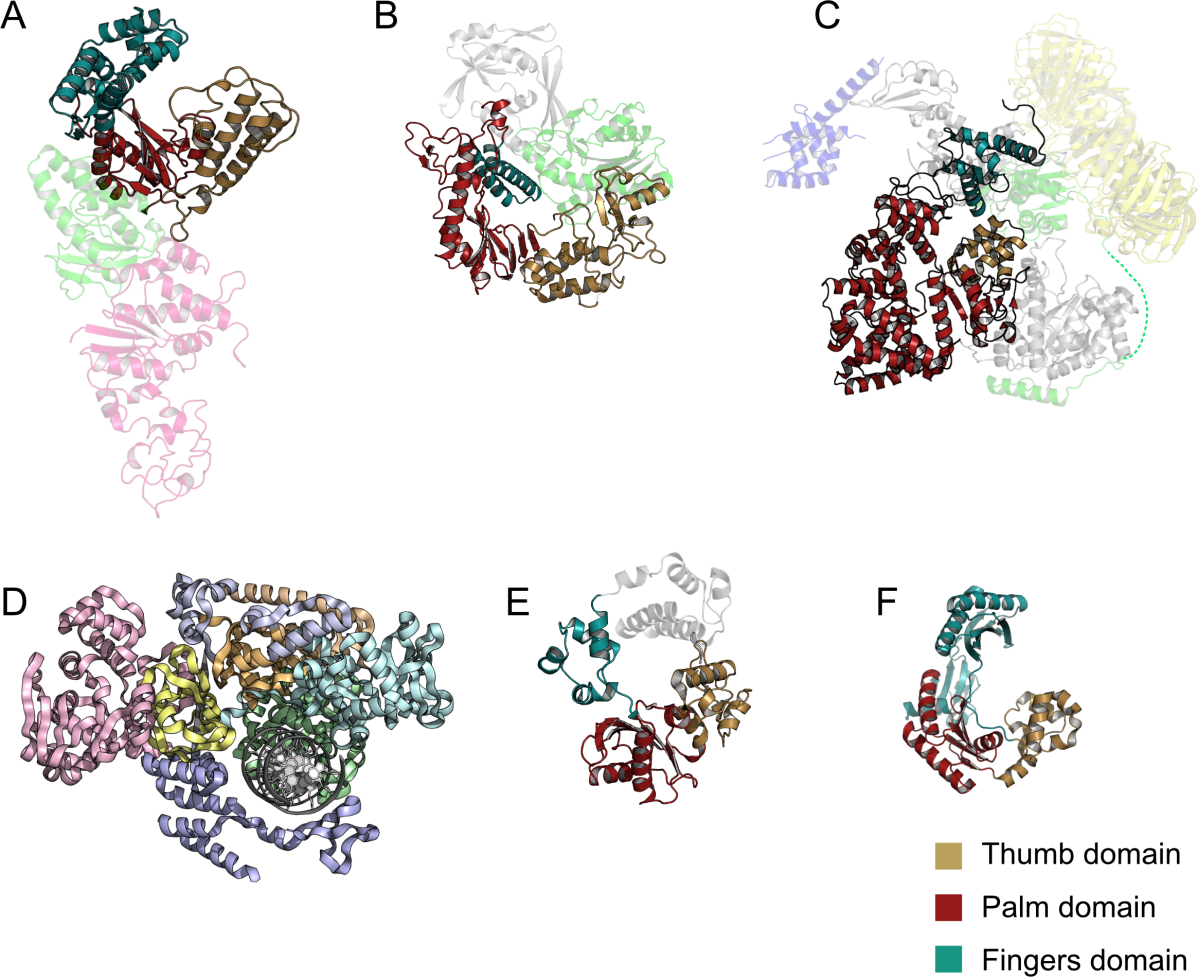
Representative DNA polymerase enzymes from each family, coloured by functional domains. (**A**) Family A: Okazaki-sealing Polymerase I from *Thermus aquaticus* (1TAQ). (**B**) Family B DNA polymerase: polymerase from *Thermococcus gorgonarius* (1TGO). (**C**) Family C DNA: Main replicative Polymerase III from *Escherichia coli* (5FKV). (**D**) Family D polymerase: DP2 subunit from *Pyrococcus abyssi* (6HMS). (**E**) Family X: Polymerase lambda from Homo sapiens (1XSP). (**F**) Family Y: DNA repair polymerase Dpo4 from *Sulfolobus solfataricus* (1S0M). Functionally equivalent domains that are found in all families except Family D are ‘Fingersfingers’ (cyan), ‘Palmpalm’ (red) and ‘Thumbthumb’ (gold) while additional regions of the main polymerase enzyme are coloured grey. Other domains with autonomous function include the 3′ to 5′ exonuclease (proofreading) domain of families A, B and C (green), the 5′ to 3′ (primer-removal domain) of PolI (light pink) and the beta sliding clamp and tau domains of Pol III (yellow and blue respectively). Regions of the structures that do not form part of the ‘right hand’ polymerase scaffold are shown as translucent to better visualise the conserved architecture. As tThe Polymerase D family enzymes lack this ‘right hand’ structure and domains are coloured arbitrarily. Figures were prepared using PyMol from experimentally- determined protein structures with coordinates available in the protein data bank (PDB) under the corresponding codes.

All DNA polymerases, except the archaeal Class D, have a broadly conserved domain architecture which resembles a right hand [53] (Figure 3). The active site is situated in the ‘palm’ domain and contains two divalent cations, usually magnesium or manganese, coordinated by a pair of carboxylate side chains. Upon binding to the primer-template duplex, the mobile ‘thumb’ domain hinges into the minor groove, forming a tight binary complex interaction. The incoming dNTP is recruited via base pairing with the template. The ‘finger’ domains interrogate the geometry of this ternary complex for the correct base pairing and position the incoming dNTP for catalysis, ensuring that only a nucleotide complementary to the template is added [53]. Formation of the new phosphodiester bond between the 3′-hydroxyl of the primer (or preceding nucleotide) and the 5′-phosphate group of the incoming nucleotide begins when the nucleotide-binding metal ion coordinates the triphosphate moiety of the incoming dNTP, positioning it for nucleotide binding. The second catalytic metal ion in the active site lowers the pKa of the 3′-hydroxyl group on acceptor strand and positions it for inline nucleophilic attack on the α-phosphate group of the incoming dNTP. The subsequent pentacoordinated bipyrimidal α-phosphate transition state is resolved by the release of PP_i_ and incorporation of the nucleotide [53–56]. Recent time-resolved crystallography studies have demonstrated participation of a third metal ion in some DNA polymerase mechanisms, where it binds immediately prior to phosphoryl transfer, lowering the energy of this step. This metal ion is not coordinated by the polymerase active site, and its recruitment has been suggested to be rate-limiting in catalysis [57,58].

Despite this conserved catalytic mechanism and ‘right hand’ core architecture, there is considerable variation in the amino acid sequences and structural topologies among DNA polymerases which has been reviewed extensively [59–64] and is depicted in Figure 3. Within the Family A, B, C, X and Y classes of DNA polymerase, there is diversity in the folds of the core thumb, palm and fingers domains, as well as the presence of additional domains and subunits. These additional regions determine the enzymes’ processivity; a measure of how many nt are added sequentially per binding event, and its fidelity; the average rate of misincorporation of the incorrect nucleotide in an insertion event. The archaeal PolD polymerases are heterodimeric, containing a small 3′-5′ proof-reading exonuclease, and the larger catalytic subunit, DP2 (Figure 3D). DP2 does not exhibit the typical ‘right hand’ topology of other DNA polymerases and is considered to be representative of a new structural class which actually shares more structural homology with some RNA polymerases [65].

These different structural classes of polymerase also have varied taxonomic distributions and functions as summarised in Table 1 and described in detail in other reviews [59–64]. Briefly, Family A polymerases include the bacterial polymerase I (PolI) enzyme as well as the polymerase subunit of the mitochondrial Pol γ and many viral polymerases. Family B polymerases comprise replicative polymerases of archaea and eukaryotes as well as bacterial PolII, which is involved in the DNA damage response of bacteria [63,66]. Family C is restricted to bacteria and provides the polymerase functionality to the PolymeraseIII (PolIII) holozyme, while Family D polymerases are unique to archaea where they participate in replication, though the details are not yet fully known [67]. Finally, Families X and Y polymerases primarily mediate translesion synthesis (TLS) and are found in all domains of life [60,68,69]. The wide range of polymerase structures and collection of appending subunits and domains reflect the diversity of biological requirements for DNA synthesis. The polymerase subunit of the replisome conducts the majority of primer extension during DNA replication and has both high processivity and fidelity to ensure accurate copying of genomic material [70,71]. Processivity is imparted by interaction of the polymerase subunit with the ring-shaped-β-sliding clamp which encircles the DNA duplex [72,73]. Fidelity is enforced by a combination of accurate nucleotide insertion during catalysis and proofreading [74] by a 3′–5′ exonuclease domain which is either encoded on the same polypeptide as the polymerase functionality (Pol I and Pol B Figure 3A and B shown in green) or as a separate subunit (Pol III Figure 3 shown in green) [75,76]. The PolI enzyme which assists with Okazaki fragment maturation as well as participating in high-fidelity DNA repair and recombination exhibits lower processivity than its replisome counterpart PolIII, and also possesses a unique 5′ to 3′ exonuclease domain for RNA primer removal [71]. In contrast to replicative polymerases, TLS polymerases, which are capable of replicating through damaged DNA typically, have both low fidelity and processivity as a consequence of structural features in the DNA binding region which allow them to bypass lesions [60,77]. Due to the mutagenic potential of this error-prone DNA synthesis, the expression of TLS polymerases is tightly regulated in all organisms [78,79].

**Table 1:** Families of DNA polymerases classified by structure.

Family	Viral	Bacteria	Archaea	Eukaryotes	Example structure
**A**	T3 DNA pol	DNA pol I ^(i, ii)^		DNA pol γ ^(I, mitochondria)^	1TAQ *T. aquaticus*
	T5 DNA pol			DNA pol θ ^(iii)^	
	T7 DNA pol			DNA pol υ ^(iii)^	
**B**	T4 DNA pol	DNA pol II ^(ii)^	DNA pol BI ^(i, ii)^	DNA pol α ^(i)^	1TGO *Thermococcus gorgonarius*
			DNA pol BII ^(i, ii)^	DNA pol δ ^(i)^	
			DNA pol BIII ^(i, ii)^	DNA pol ε ^(i)^	
				DNA pol ζ ^(iii)^	
**C**		DNA pol III ^(i)^			5FKV *E. coli*
		DNA pol E ^(i, *Bacillus subtilis*)^			
**D**			DNA pol D^(i)^		6HMS *P. abyssi*
**X**	ASFV^c^ DNA pol	DNA pol X ^(iv)^	DNA pol X ^(iv)^	DNA pol β ^(ii)^	1XSP *Homo sapiens*
				DNA pol λ ^(ii, iii)^	
				DNA pol µ ^(iii, iv)^	
				DNA pol σ ^(iii)^	
				TdT ^(iv)^	
**Y**		DNA pol IV ^(iv)^	Dpo4 DNA ^(iv)^	DNA pol η ^(iii)^	1S0M *S. solfataricus*
		DNA pol V ^(iv)^	Dbh DNA ^(iv)^	DNA pol κ ^(iii)^	
				DNA pol ι ^(iii)^	

Representative examples are given for each taxonomic kingdom as well as viruses. For additional information, we direct the reader to comprehensive reviews on structural and taxonomic classifications [59–64]. Polymerases are denoted as being involved in (i) DNA replication, (ii) High-fidelity DNA repair and recombination, (iii) TLS, (iv) Template-independent extension.

### DNA polymerases for nucleic acid amplification

Due to their monomeric structure and high fidelity, most polymerases used in biotechnology are bacterial Family A PolI enzymes and archaeal Family B PolB enzymes [80,81]. Thermophilic polymerases are required for conventional themocycled PCR applications, and while KlenTaq, the Klenow fragment of *T. aquaticus* PolI is still widely used, higher fidelity enzymes such as PolB from *Pyrococcus furiosus* have superseded it in many synthesis applications [82]. Isothermal amplification, which uses strand displacement by the polymerase rather than heat to melt the DNA duplex, has employed mesophilic polymerases such as the PolI Klenow fragment of *Geobacillus stearothermophilus* (Bst-Pol) [83]. Meanwhile, the ability to synthesise past lesions and the high error rate of TLS polymerases have been utilised for amplification of damaged DNA templates and introduction of random mutations for directed evolution [84].

Protein engineering strategies have been used to further tailor the properties of these natural polymerases. For example, fusion with additional DNA binding domains has been used to overcome the low inherent processivity of PolI and PolB [85,86]. Other properties such as salt and inhibitor tolerance have also been improved by rational design and random mutagenesis [87–89]. The ability of polymerase enzymes to amplify their own genes has made them especially amenable to directed evolution strategies, including compartmentalised self-replication (CSR) which enables the enzyme to replicate only its own encoding gene, giving rise to different variants. This method has produced KlenTaq polymerase mutants with increased thermostability and heparin resistance [90], as well as increased DNA binding affinity and resistance to inhibitors present in blood [91]. Additionally, various *Thermus* DNA polymerases have evolved capable of extending quadruple DNA mismatches and extending past DNA lesions [92]. A subsequent method called short-patch CSR (spCSR), where polymerases self-replicate a short region (or ‘patch’) of their encoding gene, enables diversification of specific motifs. Using this method, Taq polymerase mutants were obtained which had dual substrate specificity, enabling them to incorporate single ribonucleotides and deoxyribonucleotides with similar rates [93]. Further protein engineering has now evolved Taq variants capable of interconverting between DNA and C2′-OMe-modified oligonucleotides [94].

An emerging area of polymerase-mediated nucleic acid synthesis exploits the non-templated sequential nucleotide addition carried out by the terminal transferase activity of some polymerases. The most well-known of these enzymes are terminal deoxynucleotidyl transferase (TdT), PrimPol and polymerase θ. TdT is a Family X DNA polymerase, involved in DNA repair via non-homologous end joining and its structure, function and biotechnological applications are well documented [64,95–97]. Non-templated DNA synthesis is initiated by TdT binding to a DNA primer and sequentially adding random dNTPs to the 3′-hydroxyl end of the previous nucleotide [64]. A recent innovation uses a multiplexed version of TdT-mediated, template-independent DNA synthesis to ‘print’ user-defined nt on an inkjet-like platform, presenting the possibility for in-house gene writing in the near future [98]. The use of PrimPol and polymerase θ as enzymatic tools for DNA synthesis is less reported on, although both been shown to be able to engage in template-independent transferase activity in the presence of manganese making them interesting potential candidates for use in future DNA synthesis applications [99,100].

### Polymerisation with UBP-XNAs

Polymerase-based amplification of UBP-XNA requires the enzyme to act on UBP-containing substrates with similar incorporation efficiency, fidelity and processivity to natural nucleic acids [101]. PCR reactions with natural bases can have an error rate of 10^−5^ or 10^−6^, meaning one mistake for every 100,000 to 1,000,000 incorporation events, when using a family A polymerase such as Klen-Taq with an associated 3′–5′ exonuclease activity [102]. The ability of natural DNA polymerases to replicate UBP-XNA substrates with varying success has been the subject of reviews which have analysed the structural basis for differences in UBP incorporation [28,103,104]. In general, natural DNA polymerases are more tolerant to base alterations situated in the major groove of the DNA double helix, and if the shape of the UBPs remains similar to that of the canonical base pairs [28,105]. High-fidelity *in vitro* replication of both hydrogen-bonded and hydrophobic UBPs has been described using both Family A and Family B DNA polymerases; however, achieving efficiency, fidelity and processivity at similar levels observed with natural nucleic acid substrates remains elusive [16,17,44,46,106,107]. For the hydrogen-bonded Z:P base pair, fidelity is lowered when using natural DNA polymerases due to the replacement of these UBPs with canonical G:C base pairs over sequential rounds of PCR due to mispairing [17]. With the S:B pair, approximately 10% of S bases exist in an alternate tautomeric form [108] and so mispair with the canonical base T causing an S to A transition under PCR conditions [42]. Due to their lack of hydrogen bonds, the hydrophobic UBPs do not easily mispair with natural bases; however, natural DNA polymerases are unable to incorporate sequential hydrophobic UBPs when synthesising polynucleotides. For example, at least six natural bases are needed to separate the multiple Ds:Px pairs in order to achieve efficient amplification [46] while for the 5SICS:NAM UBP, incorporation of only two adjacent unnatural bases was possible before the DNA polymerase was challenged [107]. In contrast, stretches of up to four Z:P UBPs can be incorporated by DNA polymerases with high fidelity and efficiency [109].

Engineered versions of DNA polymerases provided some progress in improving UBP-XNA amplification [110]. Evolution of the Family A KlenTaq polymerase increased the incorporation efficiency of dZ when using dP as a template; however, the evolved polymerase was less able to incorporate dP relative to dZ [111]. The nuclease-deficient, N-terminal truncated Taq mutant, TiTaq, was used in PCR reactions with a high ratio of non-natural dS and dB nt to natural nt, which significantly increased correct incorporation of the dS nt and resulted in ~96% fidelity [112]. High resolution crystal structures of polymerases interacting with UBP-XNAs, coupled with computational studies have also provided considerable insight into the requirements of engineering a truly successful UBP-XNA polymerase [101,104]. The crystal structures of a mutant KlenTaq polymerase in a pre-incorporation ternary complex with dZTP opposite dP, and in a post-incorporation binary complex revealed that for native DNA polymerases, it is the post-incorporation product binding, with Z:P in the active site which causes clashes in both the primer and template strands and challenges the DNA polymerase [113]. Therefore, evolved DNA polymerases which can overcome this complication in the post-incorporation step may be beneficial. A crystal structure of native KlenTaq DNA in a ternary complex with the UBP dDs:dPxTP showed that the UBPs pair in the active site in a coplanar conformation, very similar to that of Watson–Crick base pairs; however, the increased width and height of the UBPs result in the O-helix of the finger domain not closing as tightly, which may affect the overall incorporation efficiency [101,114,115]. Molecular dynamic (MD) simulations also offer insight into the effects various mutations could have on WT polymerases’ abilities to synthesise UBP polynucleotides. MD simulations of an evolved KlenTaq polymerase variant with four amino acid substitutions showed that compared to the WT KlenTaq, this enzyme exhibited an altered flexibility which allowed it to more appropriately position the P:Z UBP for catalysis [101,114,115]. Strikingly, all four substitutions were located outside of the active site as illustrated in Ouaray et al. [115].

## Nucleic acid ligases

### Classes of DNA ligase and their biological functions

DNA ligases catalyse the formation of a phosphodiester bond between the adjacent 5′ phosphate (5′P) and 3′ hydroxyl (3′ OH) in the backbone of double-stranded DNA. This three-step reaction is powered by a nucleotide cofactor, either adenosine triphosphate (ATP) or nicotinamide adenine dinucleotide (NAD). In the first step, the ligase enzyme self-adenylates at a conserved catalytic lysine residue, forming a covalent linkage between the ε-amino group of the amino acid side chain and the a-phosphate of AMP [116]. In the second step, the enzyme–adenylate complex binds to the nicked DNA duplex and the AMP is transferred to the 5′-PO_4_ group of the DNA break. In the third and final step, the 3′ OH group is positioned for in-line nucleophilic attack on the activated 5′-PO_4_ group of the DNA–adenylate which liberates AMP from the active site and forms a new phosphodiester bond in the backbone [116].

**Figure 4:**
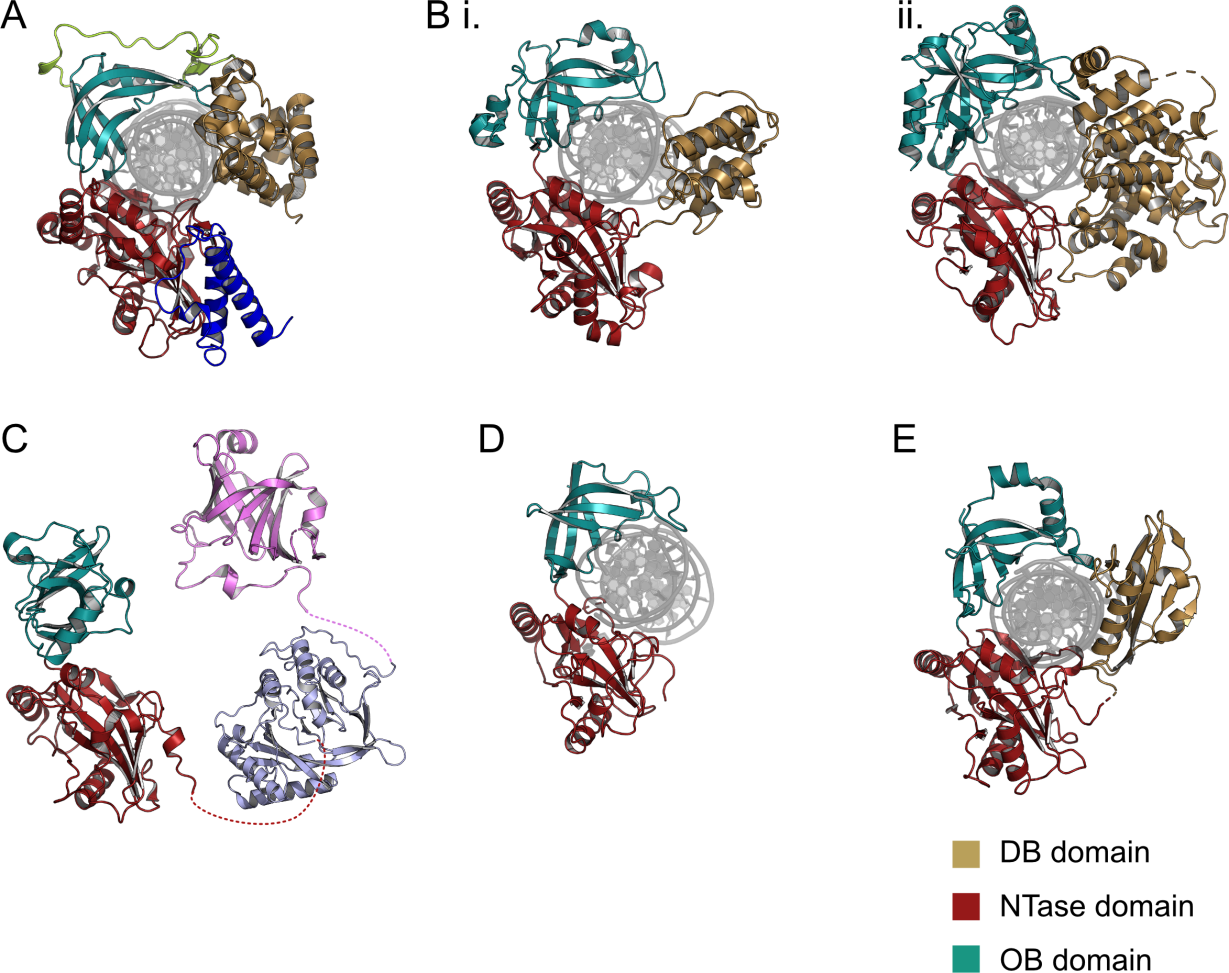
Representative DNA ligase enzymes from each family, coloured by functional domains; DNA binding domain (gold), NTase domain (red), oligonucleotide binding domain (teal). (**A**) Ligase A, replicative DNA ligase from *E. coli* (2owo). (**B**) Helical DNA binding domain: T4 bacteriophage DNA ligase (6dt1). (**C**) Ligase D: Double-strand break repair ligase; DNA ligase and PrimPol (light blue) subunits from *Mycobacterium tuberculosis* (6NHZ and 2IRU); phosphoesterase subunit (pink) from *Pseudomonas aeruginosa* (3N9B). (**D**) Minimal structure DNA ligase without DNA binding domain, Ligase E from *Alteromonas mediterranea* (6GDR). (**E**) Ligases without structural classification; African Swine Fever virus with beta -sheet DNA binding domain (6IMN). Figures were prepared using PyMol from experimentally- determined protein structures with coordinates available in the (PDB) under the corresponding codes.s.

DNA ligases are centrally involved in DNA recombination, repair and replication, all of which require the formation of new phosphodiester bonds in the DNA duplex [117]. DNA ligases can be categorised as either NAD-dependent or ATP-dependent based on the cofactor they utilise during step 1 enzyme activation [116]. Both forms have a catalytic core that consists of nucleotidyl transferase (NTase) domain and an oligobinding (OB) domain, joined by a flexible linker that enables conformational changes (Figure 4) [116,118,119]. The NTase domain, also known as the adenylation domain, contains the ligase active site including the catalytic lysine residue in the conserved KxDGxR motif. The OB domain is essential for binding the substrate DNA and rotates 180° around the interdomain linker region during engagement, inserting into the major groove to distort the duplex for catalysis [116,118,120]. NAD-dependent isoforms possess highly conserved appending domains in addition to this conserved core architecture, including a specialised Ia domain for NAD utilisation (Figure 4A, blue) as well as Zinc-finger (Figure 4A, green), BRCT and helix-turn-helix domains for DNA binding [121]. In contrast, ATP-dependent DNA ligases vary widely in their size and domain composition [118,120,122]. The simplest functional DNA ligases are of the ATP-dependent class and comprise only the core NTase and OB domains including many bacteriophage and viral isoforms [123,124] as well as the recently described Ligase-E class found in a wide range of proteobacteria [118]. The most common ATP-dependent DNA ligase architecture however has an alpha-helical domain N-terminal to the NTase domain, which assists with DNA binding and ligation efficiency (Figure 4B(i) and (ii)).

ATP- and NAD-dependent ligase isoforms have distinct taxonomic distributions with the NAD-dependent Lig-A enzyme being almost entirely restricted to bacteria where it is the essential ligase that joins Okazaki fragments during DNA replication (Table 2). Archaea and eukaryotes by contrast exclusively use ATP-dependent DNA ligases for replication. In some cases, a single ligase isoform is used for both DNA repair and replication; however, many organisms encode one or more specialised DNA repair ligases in their genomes which are usually of the ATP-dependent class. The most notable example of these are the multi-functional ‘Lig-D’ repair enzymes widely distributed among bacteria which possess auxiliary enzymatic domains with autonomous polymerase or nuclease activities that repair damaged DNA ends in advance of their re-ligation (Figure 4C, light blue and pink, respectively).

**Table 2:** Families of DNA ligase classified by structure.

Family	Viral	Bacteria	Archaea	Eukaryotes	Example structure
NAD-dependent	*Am*EPV (*amsacta moorei* entomopoxvirus)	Lig A^(i)^	LigN^(i)^ (*Haloferax volcanii*)	–	*E. coli* lig A 2owo [121]
Helical DBD	T4 DNA ligase (*Enterobacteria phage T4*)	Lig B^(ii)^	Replicative ligase^(i)^	Lig I^(i)^ Lig III^(i)^ Lig IV^(ii)^	T4 DNA ligase 6DT1 [125] Human lig I 1 × 9N [126]
Lig D	–	Lig D1^(ii)^ Lig D2^(ii)^ Lig D3^(ii)^	–	–	Ligase domain from *M. tuberculosis* 6NHZ [127] Phosphoesterase domain from *P. aeruginosa* 3N9B [128] PrimPol from *M. tuberculosis* domain [129]2IRU
Minimal	*Chlorella* virus-ligase	Lig C^(ii)^ Lig E	–	–	*A. mediterranea* Lig E 6GDR[118]
Unclassified	Swine fever virus	Lig W (*Prochlorococcus marinus*)	–	–	African swine fever virus ligase 6IMN [130]

Representative examples are given for each taxonomic kingdom as well as viruses. For additional information, we direct the reader to comprehensive reviews on structural and taxonomic classifications [116,118–120]. Ligases are denoted as being involved in (i) DNA replication or (ii) DNA repair.

### Use of ligases in DNA assembly and synthesis

The discovery of the DNA ligase was foundational for developing recombinational DNA production via traditional restriction–ligation cloning protocols, with the T4 DNA ligase being the most widely used [131]. DNA ligases remain central to assembly protocols such as Golden-Gate Assembly (Type IIS cloning) and Gibson assembly (isothermal assembly) to join the backbones of annealed fragments. The advantage of both Golden-Gate and Gibson relative to traditional restriction–ligation is that a single reaction can assemble large number of fragments (up to 15 with Gibson and 35 with Golden-Gate) into constructs of several hundred kilobases in a ‘scarless’ manner which avoids disruption of sequences features by vestigial restriction sites [132,133]. Key to the performance of the DNA ligases in both methods is the balance between bias (preference for ligating some sequences over others), fidelity (likelihood of joining mismatched ends) and efficiency [134,135]. For example, comparison of T4 and T7 DNA ligases found that while the latter produced a higher number of correct Golden-Gate assemblies, its bias against joining some sequences and its overall lower activity made the former a more suitable choice in this application [136]. A further variation on these assembly methods, the ligase cycling reaction allows direct splicing of DNA duplexes by employing a thermostable DNA ligase and a short bridging oligonucleotide. This generates a ligatable nicked intermediate when annealed to heat-denatured duplex parts and has the advantage of allowing separate gene blocks to be joined without requiring addition of extensions or homologous overhangs [137,138].

Besides assembly, ligation-mediated de novo synthesis of gene-length DNA duplexes has been explored by isothermal ligation of short octameric synthetic duplexes. The duplexes are generated by annealing 5′ phosphorylated oligonucleotides in a sequence-specific manner that leaves 4 nt ligatable overhangs resulting in products of almost 100 nt [139]. A ligation-based approach has also been used to synthesise functionalised polynucleotides, nt containing varying substituents on their nitrogenous bases. Here, an approach similar to PCR was utilised with an extendable primer annealed to a template strand which directed primer extension. Functionalised trinucleotides with a 5′ phosphate were then ligated to the primer terminus in a template-directed manner, resulting in a functionalised strand built on the natural complement. Further, this functionalised strand could be PCR amplified using DNA polymerase, enabling the enrichment of particular sequences under some selection pressure such as substrate binding [140].

### Ligation with UBP-XNAs

The use of DNA ligases to synthesise or assemble UBP-XNA has been less explored relative to the extensive work done with DNA polymerases with these substrates. Early studies on the feasibility of DNA repair involving hydrogen-bonded UPBs demonstrated that both Taq and DNA T4 ligases were able to join DNA with consecutive S:B base pairs, although the latter was more efficient [141]. T4 DNA ligase has also been shown to join single hydrophobic pairs including the Ds:Pa combination with extremely high selectivity in both a single-stranded nick and a double-stranded cohesive overhang context [142]. Recently, an innovative strategy for reading and writing DNA containing expanded base-pair alphabets was developed which utilised T4-mediated ligation as a key step in XNA synthesis [143]. Here, untemplated tailing using the small exo fragment of DNA PolI generated cohesive single UBP overhangs which were then ligated to form the new duplex. This approach was used to introduce a range of individual hydrogen-bonded UBPs into a natural sequence context, which were then able to be sequenced using nanopore technology. Crucially, several of the UBPs introduced enzymatically are intractable to oligonucleotide incorporation via phosphoramidite synthesis due to their chemical lability. This process is, in principle, amenable to the addition of multiple UPBs via iterative rounds of cleavage, tailing and re-ligation, provided the enzymes involved will tolerate consecutive tracts of UBPs.

The potential of DNA ligases to deliver XNA with derivatives on sugar moiety has been widely examined and provides an example of the utility of this approach which may also be applied to UBP-XNA [51,144]. Commercially available DNA ligases T3, T4 and T7 were demonstrated to join nicked duplexes containing TNA and 2′OMe RNA, with the latter XNA also being ligated by the *Chlorella* virus (SplintR) enzyme [51,144]. All enzymes showed a marked bias against DNA-XNA joins with the XNA strand in the donor position providing the 5′ phosphate to the join relative to the converse XNA-DNA acceptor–donor configuration. Among this small panel, the T4 DNA ligase proved the most effective and was demonstrated to ligate a wide range of backbone-XNA substrates, including 2′-fluoroarabino nucleic acids (FANA), 1,5-dianhydrohexitol nucleic acids (HNA) and locked nucleic acid (LNA) in the presence of crowding agents Pe.g.8000 and betaine [144]. Beyond optimisation of buffer, crowding agents and cofactor concentrations, attempts have been made to improve the enzymatic ligation of modified backbone XNAs by rational engineering. The *Chlorella* virus ligase was selected as a scaffold to improve joining of hexitol-based XNA nt (HNA) based on its small size and well-characterised structure. Computational prediction of the XNA-ligase complex indicated that lengthening the inter-domain hinge region would better accommodate the wider diameter of the HNA duplex and guided the insertion of a single glycine residue in the interdomain linker which was then able to ligate various combinations of 2′OMe RNA and HNA, unlike wild-type controls [145]. Other modifications such as fusion of processivity domains to otherwise wild-type enzymes have proved less effective in increasing backbone-XNA ligation [144].

## The future of UBP-XNA-joining enzymes and concluding remarks

Despite the considerable advances in synthesising and characterising XNA with expanded base-pair alphabets, many of the key enzymes in the conventional molecular biologist’s toolbox exhibit sub-par performance when challenged with XNA containing UBPs. To provide superior UBP-XNA joining enzymes, we argue for an integrated discovery cycle that explores the UBP-XNA compatibility of DNA ligases and polymerases from existing biological diversity, coupled to further engineering-based improvements.

Current UBP-XNA compatible enzymes have been largely based on conventional molecular biology enzymes; in many examples employing the commercially available version as a source. While this is a logical starting point to build from, it means that much of the structural and functional diversities among these enzymes classes remain unexplored in the context of UBP-XNA enzymology. For example, most polymerases used in molecular biology are derived from the PolA and B families and most UBP-XNA tools are variants of the KlenTaq enzymes; likewise, all XNA ligation (backbone and UBP) has focused on a narrow range of phage enzymes with most work being on the T4-DNA ligase. However, as described above, there is an enormous diversity of ligase scaffolds found in nature, many of which have only been minimally studied, especially in the context of UBP-XNA.

Recombinant enzymes spanning the natural polymerase/ligase diversity need to be empirically and systematically tested with a range of standardised UBP-XNA substrates and the results mapped back to their sequence and structural features. This will be essential to build a better picture of which features promote or inhibit UBP-XNA joining, and inform future discovery initiatives, as well as provide guidelines to rational engineering endeavours. Catalytic benchmarks should include not only the efficiency of amplification/ligation but also quantify fidelity and bias as these will have a direct bearing on the suitability of the enzymes in different applications. It is important that such benchmarking is undertaken using conditions that reflect those used in the UBP-XNA assembly applications and take account of factors such as sequence bias. In this regard, assays utilising sequence read-out that can be conducted in a multiplex format such as those used to profile DNA ligase fidelity would prove useful [134–136]. The replication machinery of extremophilic microbes represents another possible avenue for discovering novel molecular biology enzymes, which may have a particular benefit to UBP-XNA compatibility. Among the DNA polymerases and ligases described above that join UBP substrates, the majority are meso- and thermophilic, leaving psychrophilic variants relatively under-explored. In part, this reflects the fact that truly psychrophilic enzymes tend to be difficult to characterise in laboratory settings due to their innate instability and heat-lability. However, psychrophilic enzymes also hold biotechnological and industrial value [146,147], especially, in the case of polymerases, if they can be engineered to perform strand separation at low temperatures as this allows them to be used in isothermal amplification protocols [148].

We would also argue that although considerable progress has been made in improving polymerase performance with UBP-XNAs, ligation-based strategies such as those demonstrated for modified backbone XNAs [51,144] should be more thoroughly explored. This could be accomplished either as the sole step of UBP-XNA synthesis using approaches akin to Kawabe et al*.* [143] or by assembly of longer tracts which have been amplified by a UBP-XNA-compatible polymerase. This would overcome limitations in current polymerase enzymes towards insertion of multiple UBP bases. Implementing such a strategy will, however, require engineering endeavours for DNA ligase enzymes on-par with those directed, to date, more thoroughly at the DNA polymerase enzymes. In the case of the DNA ligases, a structure-centric approach will be necessary as there are currently no simple cell-free directed evolution protocols for DNA ligases that are amenable to incorporation of UBP-XNAs. Ideally, UBP-XNA-ligase engineering will use experimentally determined structures of the enzyme-XNA complex or alternately enzyme-DNA on which the UBP-XNA can be modelled and then analysed in molecular dynamics simulations. Although advances in structural modelling using artificial intelligence have recently been extended to prediction of protein-nucleic acid complexes [149,150], these are unable to model modifications or reaction intermediates, and are known to produce spurious results which could limit their utility in structure-guided engineering.

Finally, to realise the true potential of UBP-XNAs, the compatible enzymology must extend beyond UBP-XNA joining to include other activities as well. For example, incorporating UBPs to generate synthetic/semi-synthetic organisms will require either compatibility of the entire replication machinery including helicases, topoisomerases, initiation and termination factors with the unnatural bases or supplementation of the native enzymes with co-expressed UBP-XNA compatible homologs. Furthermore, incorporation of additional amino acids into an expanded genetic code will require tRNA synthetases capable of charging UBP-XNA-containing anticodons with unnatural amino acids. For stable error-free replication, damage and mismatch repair systems will be necessary and could also improve the accuracy of synthetic UBP-XNA. Arguably one of the most important emerging applications for XNA is the deployment of ribo-XNA for pharmaceutical applications. Although this review has focused on the 2′-deoxy-derivatives of these polymers, the central role of the modified N1-methyl-pseudouridine nucleotide in decreasing immunogenicity of the Covid19 mRNA vaccines [22,23] underscores the utility of XNAs. It also seems likely that enzymes replicating and ligating ribo-XNA will represent the next frontier of this area.

## Supplementary material

Figure S1.

## Abbreviations

AEGIS: Artificially Expanded Genetic Information Systems
ATP: adenosine triphosphate
BPNA: boranophosphate
CSR: compartmentalizsed self-replication
DMT: dimethoxytrityl
FANA: fluoroarabino nucleic acids
HNA: hexitol nucleic acid
LNA: locked nucleic acid
MD: Molecular dynamic
NAD: nicotinamide adenine dinucleotide
NTase: nucleotidyl transferase
OB: oligobinding
PDB: protein data bank
PolI: polymerase I
PolIII: PolymeraseIII
SELEX: systematic evolution of ligands by exponential enrichment
TLS: translesion synthesis
TNA: threose nucleic acid
TdT: terminal deoxy nucleotidyl transferase
TiEOS: Template -independent enzymatic oligonucleotide synthesis
UBP-XNAs: unnatural nucleobase pairs
XNA: Xenobiotic Nnucleic Aacid
dNTPs: deoxy ribonucleotide triphosphates
nt: nucleotides
phNA: phosphonate nucleic acid
spCSR: short-patch
