# Supplementary figures and images for: Nucleic acid joining enzymes: biological functions and synthetic applications beyond DNA
Source: Biochem J. 2025 Jan 22;482(2):BCJ20240136. doi: 10.1042/BCJ20240136 (PMC12133292; doi:10.1042/BCJ20240136)

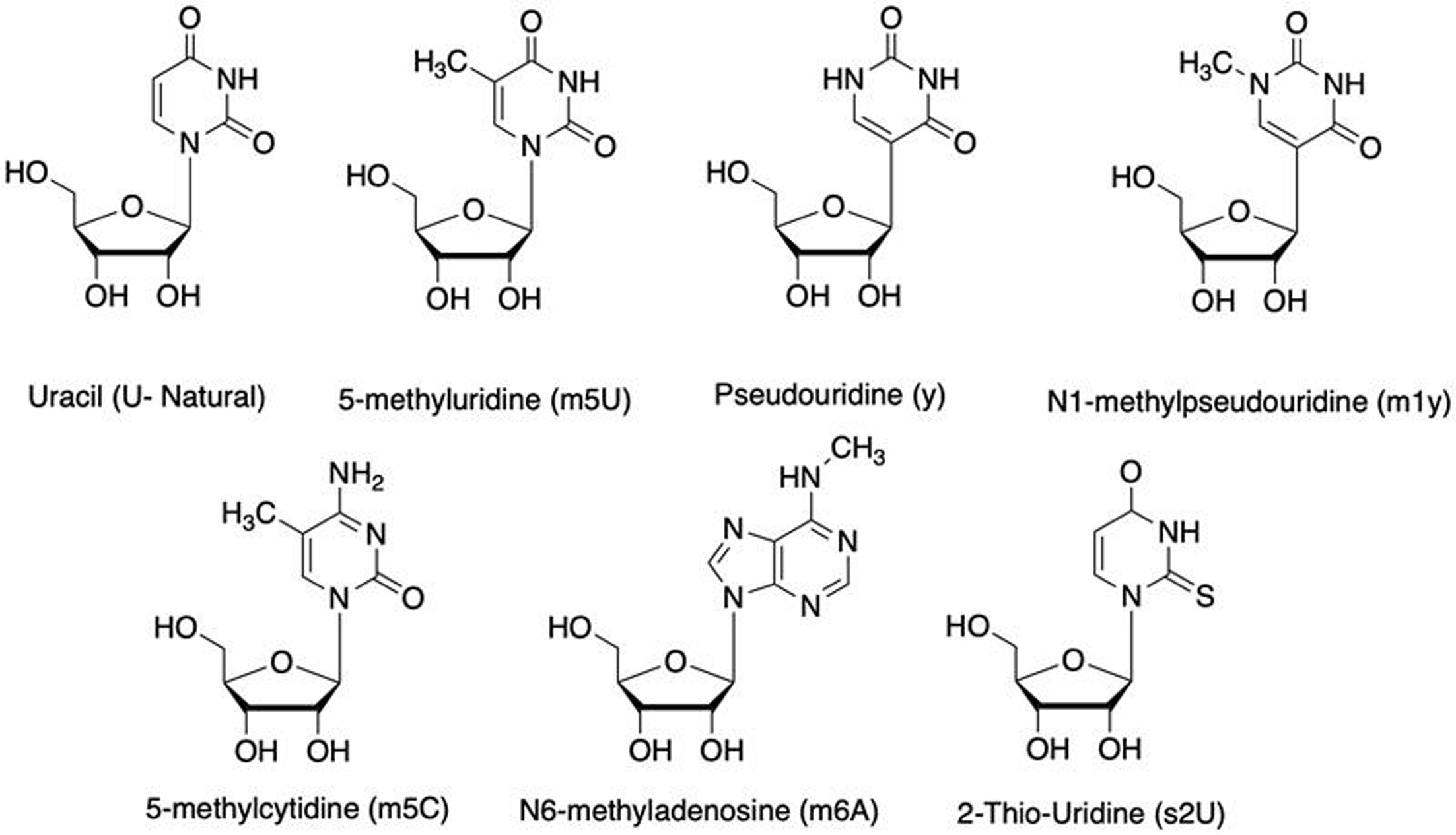

Supplement: Figure S1. [file bcj-482-2-BCJ20240136-s001.tif]
